# Atypical Ocular Myasthenia-Like Presentation in a Patient With a Heterozygous SQSTM1 Variant

**DOI:** 10.7759/cureus.87371

**Published:** 2025-07-06

**Authors:** Milaris M Sanchez-Cordero, Josean M Rosado, Felix Rivera Troia, Fernando J Ocasio Villa

**Affiliations:** 1 Internal Medicine, Mayaguez Medical Center, Mayaguez, PRI; 2 Genetics, Mayaguez Medical Center, Mayaguez, PRI

**Keywords:** genetic testing, neuromuscular junction disorder, ocular myasthenia, repetitive nerve stimulation, seronegative myasthenia gravis, sqstm1

## Abstract

We report the case of a patient presenting with diplopia, ptosis, and blurred vision who was found to have a heterozygous SQSTM1 gene variant (c.1175C>T) following electrophysiological evidence of a postsynaptic neuromuscular junction disorder. The patient had negative antibody testing, mildly elevated creatine phosphokinase (CPK), and abnormal organic acid analysis. Repetitive nerve stimulation demonstrated a decremental response consistent with impaired postsynaptic transmission. Given the atypical presentation and systemic findings, genetic testing was pursued, revealing a variant of uncertain significance in SQSTM1, despite the variant being classified as pathogenic in genetic databases. This case raises the possibility of a broader neuromuscular phenotype associated with SQSTM1 mutations and underscores the value of genetic testing in seronegative neuromuscular disorders. It also highlights the need to consider SQSTM1 variants in the differential diagnosis of seronegative neuromuscular presentations.

## Introduction

Myasthenia gravis (MG) is a chronic autoimmune neuromuscular disorder characterized by fatigable skeletal muscle weakness. Ocular symptoms such as diplopia, ptosis, and blurred vision are common early manifestations and may remain isolated in a subset of patients with ocular MG [1]. Diagnosis relies on clinical examination, antibody testing (e.g., acetylcholine receptor (AChR), muscle-specific kinase (MuSK)), and neurophysiological studies, such as repetitive nerve stimulation (RNS) and single-fiber electromyography [2,3]. However, up to 15% of cases are seronegative, complicating the diagnostic approach and prompting the investigation of alternative or coexisting genetic contributors [4]. While MG is classically autoimmune in nature, genetic and metabolic contributors are increasingly recognized in atypical or seronegative cases. These cases warrant broader diagnostic strategies, including the use of comprehensive gene panels.

The SQSTM1 gene encodes sequestosome 1 (p62), a multifunctional adaptor protein involved in key cellular processes such as selective autophagy, oxidative stress response, and protein aggregate degradation [5]. Pathogenic variants in SQSTM1 have been most commonly linked to Paget's disease of bone, amyotrophic lateral sclerosis (ALS), and frontotemporal dementia (FTD) [6-8]. More recently, mutations have been identified in patients with complex neurodegenerative or myopathic phenotypes, suggesting broader roles for SQSTM1 in neuronal maintenance, mitochondrial quality control, and synaptic integrity [9-11].

We describe a patient with progressive ocular neuromuscular symptoms and RNS findings consistent with a postsynaptic transmission defect, who was found to carry a heterozygous SQSTM1 variant (c.1175C>T). This case highlights the potential involvement of SQSTM1 in neuromuscular transmission disorders and raises questions about its phenotypic spectrum.

## Case presentation

**Figure 1:**
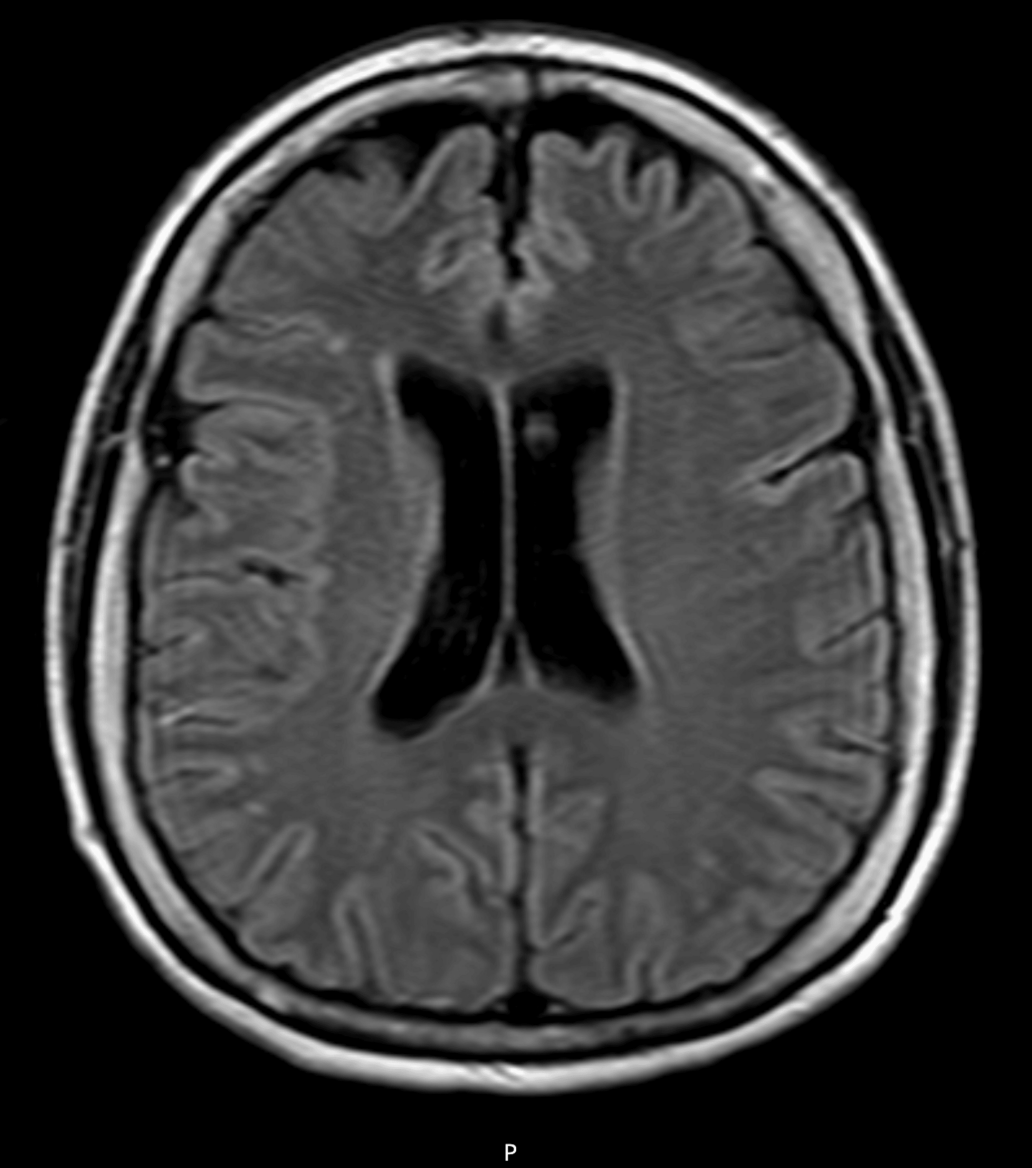
MRI of the head/brain without contrast The ventricles and sulci are normal as to size, shape, and configuration for the patient's age. There are minimal scattered foci of T2 prolongation/FLAIR hyperintense signal within the supratentorial cerebral white matter, which are nonspecific though probably represent a sequela of chronic microangiopathic changes. MRI: magnetic resonance imaging; FLAIR: fluid-attenuated inversion recovery

A 64-year-old woman presented with a six-month history of progressive diplopia, ptosis, and blurred vision. Neurological examination confirmed fatigable bilateral ptosis and impaired extraocular movement. There was no facial weakness, dysarthria, or generalized limb weakness (Figure 1).

Laboratory evaluation revealed a mildly elevated creatine kinase (183 U/L, normal 30-135) and alkaline phosphatase. An organic acid panel showed elevated lactic acid (133 mmol/mol creatinine, normal 0-50), pyruvic acid (25, normal 0-15), 3-hydroxybutyric acid (34, normal 0-4), and acetoacetic acid (9, normal 0-4). Serum carnitine (total and free), amino acids, aldolase, rapid plasma reagin (RPR), fibrinogen, D-dimer, HIV 1/2, antinuclear antibodies (ANA), vitamin B12, thyroxine (T4), free triiodothyronine (FT3), thyroid-stimulating hormone (TSH), and lipid panel were within normal limits. The electroencephalogram (EEG) was also normal (Table 1).

**Table 1:** Laboratory results MCV: mean corpuscular volume; PT: prothrombin time; INR: international normalized ratio; PTT: partial thromboplastin time; ANA: antinuclear antibodies; ASMA: anti-smooth muscle antibodies; TIBC: total iron binding capacity; AFP: alpha-fetoprotein; RPR: rapid plasma reagin; FEU: fibrinogen equivalent units

Parameter	Result	Reference range
Hemoglobin	15.60 g/dL	11.20-15.20 g/dL
Hematocrit	46.40%	33.60-45.60%
MCV	94 fl	81-99 fl
PT	14 seconds	11.80-17.20 seconds
INR	1.04	0.8-1.2
PTT	26.90 seconds	19.20-37.60 seconds
ANA	Negative	Negative
ASMA	Negative	Negative
Alpha-1 antitrypsin	180 mg/dL	100-190 mg/dL
Ferritin	133 ng/mL	12-150 ng/mL (women)
Iron	118 µg/dL	60-170 µg/dL
TIBC	287 µg/dL	240-450 µg/dL
AFP	5.60 ng/mL	0.00-8.00 ng/mL
Creatine kinase	183 U/L	30-135 U/L
Alkaline phosphatase	131 U/L	40-129 U/L
Lactic acid (urine)	133 mmol/mol Cr	0-50 mmol/mol Cr
Pyruvic acid (urine)	25	0-15
3-Hydroxybutyric acid	34	0-4
Acetoacetic acid	9	0-4
Serum carnitine (total/free)	Normal	Varies by lab, typically: total 25-50 µmol/L, free 20-40 µmol/L
Aldolase	2.4 U/L	1.0-7.5 U/L
RPR, HIV 1/2	Non-reactive	Non-reactive
Vitamin B12	356 pg/mL	200-900 pg/mL
Lipid panel	Normal	Normal
Fibrinogen	205 mg/dL	200-400 mg/dL
D-dimer	<0.5 µg/mL FEU	<0.5 µg/mL FEU

Serologic testing for MG, including AChR binding, blocking, and modulating antibodies, was negative. Electrophysiologic testing with RNS revealed a significant decremental response in the right ulnar and facial nerves at low-frequency stimulation, consistent with a postsynaptic neuromuscular junction disorder. Given the negative antibody results and abnormal metabolic profile, genetic testing was pursued. A comprehensive germline neuromuscular panel identified a heterozygous SQSTM1 variant (c.1175C>T). No other pathogenic mutations were identified. The patient has a known history of hepatic cirrhosis secondary to metabolic dysfunction-associated steatohepatitis (MASH), but no family history of neuromuscular disease or neurodegeneration (Table 2).

**Table 2:** Genetic sequencing/analysis showing a positive result for the SQSTM1 gene mutation

Gene	Variant	Zygosity	Variant classification
SQSTM1	c.1175c>T (p.Pro392Leu)	Heterozygous	Pathogenic

## Discussion

This case presents an adult with isolated ocular neuromuscular symptoms, including diplopia, ptosis, and blurred vision. RNS showed a decremental response in the right ulnar and facial nerves, supporting a postsynaptic neuromuscular junction disorder, most consistent with MG. However, antibody panels were negative, and the patient exhibited additional systemic abnormalities, such as elevated creatine kinase, abnormal organic acid profile, and a history of MASH, raising suspicion for a multisystem or genetic condition.

Genetic testing via germline mutation revealed a heterozygous SQSTM1 variant (c.1175C>T). While this specific variant has not been previously associated with MG or neuromuscular junction disorders, SQSTM1 is increasingly recognized as a gene with broad relevance to neurodegeneration, axonal transport, and autophagy regulation [5,9,10]. The encoded p62 protein plays a central role in mitophagy, and its dysfunction may impair mitochondrial turnover and neuromuscular synaptic health [11,12].

There is growing recognition that autophagy-related genes, including SQSTM1, may act as modifiers of neuromuscular disease or contribute to atypical clinical phenotypes, particularly in patients who lack common autoantibodies or clear structural abnormalities [13,14]. Studies have shown that SQSTM1 mutations can result in complex neurological presentations, ranging from ALS and FTD to myopathy and sensorimotor neuropathy, expanding the gene's phenotypic range [6,9,14].

This discovery in a patient with a clear electrophysiologic evidence of postsynaptic dysfunction supports the need for further investigation into SQSTM1's role in neuromuscular transmission. Functional studies assessing p62 stability, lysosomal trafficking, and synaptic vesicle recycling could elucidate whether and how SQSTM1 variants contribute to transmission failure. Furthermore, this case supports the utility of comprehensive gene panels in evaluating atypical neuromuscular presentations, especially in seronegative or antibody-negative cases [4,13].

## Conclusions

This report suggests a possible association between a rare SQSTM1 variant and an ocular neuromuscular junction disorder. Future research should explore whether SQSTM1 mutations can act as primary drivers or cofactors in the pathogenesis of neuromuscular diseases. Further studies are needed to determine whether SQSTM1 mutations could serve as pathogenic drivers or modifiers in seronegative neuromuscular disorders.
